# Long-term success of low-frequency subthalamic nucleus stimulation for Parkinson’s disease depends on tremor severity and symptom duration

**DOI:** 10.1093/braincomms/fcab165

**Published:** 2021-07-28

**Authors:** Nirosen Vijiaratnam, Christine Girges, Thomas Wirth, Timothy Grover, Francesca Preda, Elina Tripoliti, Jennifer Foley, Emma Scelzo, Antonella Macerollo, Harith Akram, Jonathan Hyam, Ludvic Zrinzo, Patricia Limousin, Thomas Foltynie

**Affiliations:** 1 Department of Clinical and Movement Neurosciences, UCL Queen Square Institute of Neurology, London WC1N 3BG, UK; 2 Unit of Functional Neurosurgery, the National Hospital for Neurology and Neurosurgery, London, UK; 3 Unit of Neurology of Ospedale “M. Bufalini” of Cesena, Cesena, Italy; 4 Department of Neurology, the Walton Centre NHS Foundation Trust, Liverpool, UK

**Keywords:** Parkinson’s disease, STN-DBS, low-frequency stimulation, axial features

## Abstract

Patients with Parkinson’s disease can develop axial symptoms, including speech, gait and balance difficulties. Chronic high-frequency (>100 Hz) deep brain stimulation can contribute to these impairments while low-frequency stimulation (<100 Hz) may improve symptoms but only in some individuals. Factors predicting which patients benefit from low-frequency stimulation in the long term remain unclear. This study aims to confirm that low-frequency stimulation improves axial symptoms, and to go further to also explore which factors predict the durability of its effects. We recruited patients who developed axial motor symptoms while using high-frequency stimulation and objectively assessed the short-term impact of low-frequency stimulation on axial symptoms, other aspects of motor function and quality of life. A retrospective chart review was then conducted on a larger cohort to identify which patient characteristics were associated with not only the need to trial low-frequency stimulation, but also those which predicted its sustained use. Among 20 prospective patients, low-frequency stimulation objectively improved mean motor and axial symptom severity and quality of life in the short term. Among a retrospective cohort of 168 patients, those with less severe tremor and those in whom axial symptoms had emerged sooner after subthalamic nucleus deep brain stimulation were more likely to be switched to and remain on long-term low-frequency stimulation. These data suggest that low-frequency stimulation results in objective mean improvements in overall motor function and axial symptoms among a group of patients, while individual patient characteristics can predict sustained long-term benefits. Longer follow-up in the context of a larger, controlled, double-blinded study would be required to provide definitive evidence of the role of low-frequency deep brain stimulation.

## Teaser

Vijiaratnam et al. report beneficial and durable improvements in axial complications of subthalamic nucleus deep brain stimulation with low frequency stimulation in one of the largest cohorts reported to date. Less severe tremor and an earlier occurrence of axial complications during post-operative follow-up predicts the durability of low frequency stimulation, which is a novel finding.

## Graphical Abstract

**Graphical Abstract:**
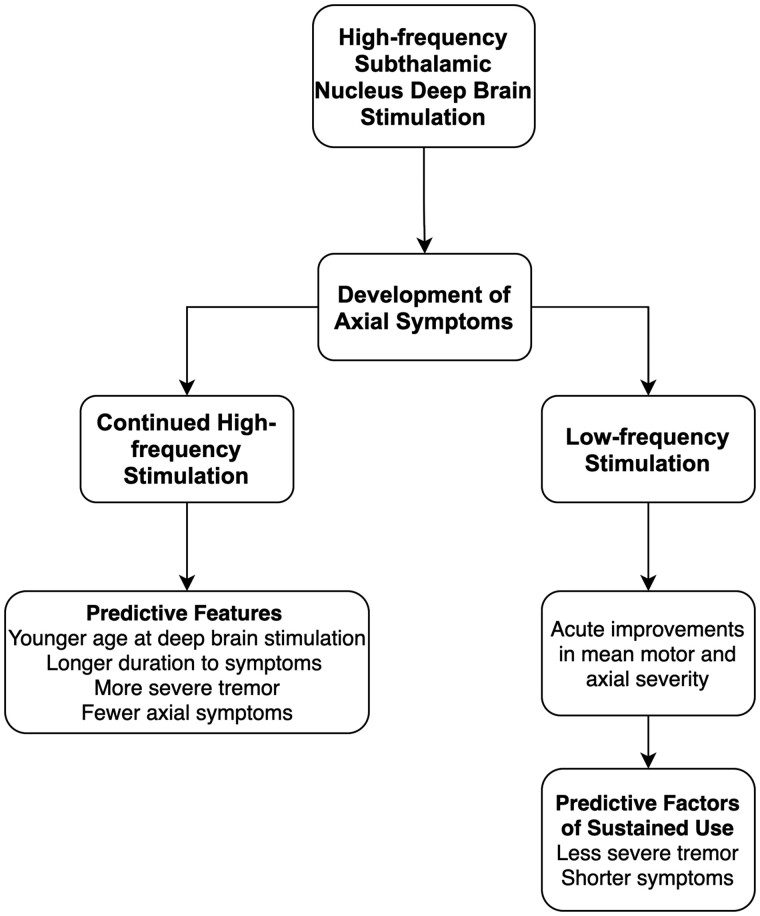

## Introduction

Deep brain stimulation (DBS) of the sub-thalamic nucleus (STN) is an efficacious treatment for motor symptoms in Parkinson’s disease.^1^ A proportion of patients can develop axial symptoms for the first time or experience their worsening following STN DBS^1^^,^^2^ although it can be difficult to distinguish the contribution of disease progression^3^ from the contribution of chronic high-frequency stimulation (HFS, >100 Hz).^4^ While low-frequency stimulation (LFS, <100 Hz) has been shown to improve axial symptoms by some teams,^5^ findings vary between studies^6^^,^^7^ and the durability of benefits remain uncertain.^8^^,^^9^

Factors contributing to these heterogeneous outcomes likely include the post-operative lead location,^2^^,^^8^^,^^10^^,^^11^ pre-operative axial symptom type and severity,^11^ the degree of brain atrophy^12^ and the responsiveness of these symptoms to dopamine pre-operatively.^11^ Here, we objectively assessed the utility of LFS in a prospective group of patients with STN DBS who developed axial features, then explored features that were associated with the clinical decision to trial LFS and the durability of its use across a larger group of patients.

## Materials and methods

Parkinson’s disease patients treated with STN DBS at the National Hospital for Neurology and Neurosurgery (NHNN), London from 2010 were assessed in this study. Patient selection, pre-operative assessments and our neurosurgical procedure have previously been described.^13^ Patients typically start stimulation on a frequency of 130 Hz and a pulse width of 60 µs. Ethical approval was provided by the local joint research ethics committee.

Parkinson’s disease patients who experienced disabling gait and speech symptoms for at least six months were recruited into this study to determine the efficacy of LFS. Patients with other medical conditions that could interfere with their gait and speech were excluded. After an assessment on their chronic HFS setting, frequency was reduced to 80 Hz and amplitude modified to maintain estimated total electrical energy delivered (TEED).^14^ No contact(s) or pulse width changes were made. Amplitude was further modified if clinically indicated. Medication changes were permitted if necessary in the subacute setting. Patients were then assessed 15–60 min after switching to optimized LFS (acute LF assessment: A-LFS) and 15 days after switching to LFS (sub-acute LF assessment: SA-LFS). Assessments were performed after overnight dopaminergic treatment withdrawal [OFF medication (MED) condition] and 1 h after a supramaximal dose of levodopa (ON MED condition). Participants were rated using the unified Parkinson’s disease rating scale (UPDRS) part III total, axial (sum of gait, speech and posture), and tremor sub-scores in the ON and OFF states. The UPDRS part II (and relevant axial sub scores) and the PDQ39 were also recorded in the ON state. Gait was assessed with the Stand–Walk–Sit (SWS) Test,^15^ the Tinetti Mobility Test (TMT)^16^ in both ON MED and OFF MED conditions and the freezing of gait questionnaire (FOG-q). The SWS test included the time (SWS time), number of steps taken (SWS steps) and the number of FOG episodes (SWS freezing episodes) occurring when a patient stands up from a chair, walks for 7 m, turns around and sits down again. A higher score depicts a more severe gait impairment. The TMT evaluates the risk of falling and includes gait and balance sub scores. The patient initially sits in an armless chair and is asked to rise up, stay standing, turn 360° and then sit back down. Next, the patient is asked to walk a few metres at a normal speed, followed by turning and walking back at a ‘fast but safe’ speed before sitting back down. Different aspects of gait and balance are assessed during this process. Gait is scored out of 12 and balance out of 16 producing a total score out of 28. The lower the score, the higher the risk of falling. The FOG-q characterizes the impact of this symptom on their day-to-day function and quality of life. A higher score represents a higher FOG burden. Speech was assessed with ‘Assessment of Intelligibility for Dysarthric Speech’ (AIDS) test.^17^ The intelligibility score derived is the percentage of words correctly transcribed after two exposures to the sentences by a native English speaker who was blinded to the conditions.

The records of all Parkinson’s disease patients who underwent STN DBS at NHNN from 2010 to the end of 2017 were then examined to identify patients that had ever been switched to LFS (frequency ≤ 100 Hz). Only cases using Medtronic Kinetra/Activa systems were selected to minimize potential variations introduced by more recent stimulation parameter options. The pre-operative UPDRS 1–4 total, axial and tremor sub-scores in the ON and OFF states and UPDRS 2 axial sub scores were noted. The dates of surgery, development of axial symptoms, switching from HFS to LFS, and/or switching back to HFS (if this occurred) and of last follow-up for both HFS and LFS patients were also noted. Patients were then divided into two groups; (i) those maintained on HFS and (ii) those who had been switched to LFS. LFS patients were then further divided into those who remained on LFS and those who reverted back to HFS (failure with LFS). Clinical features of these groups were then compared. Factors predictive of the decision to use LFS and those that limited its durability were identified.

### Statistical analysis

Clinical outcomes for continuous values are presented as mean ± SD. Numeric values were checked for normality using one-sample Kolmogorov–Smirnov and Shapiro–Wilk analysis. Continuous data comparing baseline and postoperative group scores were analysed by *t*-test if normally distributed, or Mann–Whitney test for non-parametric data. The Wilcoxon signed-rank test was used for comparison of non-parametric paired data between the three stimulation settings using Bonferroni correction to control for multiple comparisons. A Cox regression model was utilized to assess the relationship between pre-operative clinical factors and (i) likelihood of patients being switched to LFS and (ii) time duration spent on LFS. All reported *P*-values are two-sided and significance was assigned to *P* < 0.05. All data were analysed using SPSS (Release V.26.0 Chicago, IL, USA).

### Data availability

The data that support the findings of this study are available from the corresponding author, upon reasonable request.

## Results

Of 22 patients enrolled, 2 immediately reverted to HFS due to intolerable worsening of symptoms. Of the remaining 20 patients, 15 were male with an average disease duration 15.7 ± 3.9 years. Seventeen of 20 suffered from a mixture of axial symptoms, 2 pure gait difficulties and 1 solely speech deficit. Mean stimulation amplitudes were increased bilaterally in the subacute setting compared to HFS [left 3.55 V (0.81) versus 2.97 V (0.67); *P* < 0.01 right 3.53 V (0.94) versus 2.97 V (0.79); *P* < 0.01]. LFS resulted in an improvement in overall motor function in the sub-acute (UPDRS parts 2 and 3 scores) settings in the OFF medication states and UPDRS part 2 in the ON medication state (Table 1). LFS improved UPDRS axial sub-items in both the ON and OFF states in the sub-acute setting. Gait measures (SWS, TMT, FOG-q) were also improved in the ON state at the sub-acute timepoint. Speech improvements (UPDRS 3 and UPDRS 2 speech scores) were seen in the ON state at SA-LFS. Of the 20 participants, we were able to objectively quantify speech intelligibility in HFS and LFS conditions using the Speech Intelligibility test in 13 patients. Improvements in the speech intelligibility score during the sub-acute LFS OFF state assessment (74.4 ± 112.6 versus 67.3 ± 67.3; *P* = 0.02). A significant improvement in speech intelligibility was noted in both the acute and sub-acute assessments from LFS in the ON medication state. LFS resulted in an overall improvement in the total PDQ39 score which was most evident in cognition and communication subdomains.

**Table 1:** Comparison of scores between HFS and LFS across different time points

Scores	HFS Mean (SD)	A-LFS Mean (SD)	SA-LFS Mean (SD)	*P*-value (A-LFS vs HFS)	*P*-value (SA-LFS vs HFS)
OFF medication					
UPDRS 3 Total	32.9 (9.8)	31.3 (9.6)	29.5 (7.7)	0.12	<0.01
UPDRS 3 Axial	6.0 (2.4)	5.0 (2.2)	4.6 (2.3)	<0.01	<0.01
UPDRS 3 Tremor	1.2 (1.8)	1.9 (2.1)	2.1 (2.5)	0.27	0.14
UPDRS 2 Total	20.3 (6.3)		16.5 (6.9)		0.02
UPDRS 3 Speech	2.2 (0.8)	1.9 (0.7)	1.7 (0.7)	0.03	0.48
UPDRS 2 Speech	2.4 (0.8)		1.9 (1.0)		0.36
AIDS (Speech Intelligibility test)	67.3 (19.3)	76.7 (13.4)	74.4 (12.6)	0.27	0.02
SWS time	15.7 (4.4)	13.9 (3.0)	13.7 (3.8)	<0.01	0.08
SWS steps	27.4 (13.1)	24.4 (8.1)	23.1 (5.3)	<0.01	0.99
SWS freezing episodes	0.7 (1.6)	0.2 (0.7)	0.1 (0.2)	0.06	0.08
TMT balance	13.9 (1.8)	14.6 (1.7)	15.1 (1.1)	0.02	0.01
TMT gait	9.9 (2.0)	10.3 (1.6)	11.1 (0.9)	0.21	0.03
TMT total	23.8 (3.1)	24.9 (2.3)	26.2 (1.5)	0.02	<0.01
ON medication					
UPDRS 3 Total	22.4 (9.1)	19.3 (9.4)	19.3 (7.9)	0.01	0.06
UPDRS 3 Axial	5.3 (2.4)	4.0 (2.3)	3.7 (1.9)	<0.01	0.01
UPDRS 3 Tremor	0.4 (0.7)	0.8 (1.2)	0.8 (1.4)	0.11	0.72
UPDRS 2 Total	14.9 (6.7)		10.9 (6.4)		0.02
UPDRS 3 speech	2.3 (1.0)	1.7 (0.7)	1.7 (0.6)	0.02	0.02
UPDRS 2 Speech	2.1 (1.1)		1.4 (1.0)		0.06
AIDS (Speech Intelligibility test)	57.5 (13.3)	76.7 (11.6)	75.9 (12.0)	0.02	0.05
SWS time	13.3 (4.1)	11.9 (3.3)	12.0 (1.8)	<0.01	0.01
SWS steps	24.8 (11.3)	23.3 (9.3)	20.7 (3.2)	0.03	0.05
SWS freezing episodes	0.5 (1.2)	0.1 (0.4)	0.1 (0.2)	0.21	0.24
TMT balance	14.0 (2.0)	15.5 (3.0)	15.2 (1.0)	<0.01	0.01
TMT gait	10.2 (2.1)	10.5 (1.5)	11.3 (0.9)	0.69	0.03
TMT total	24.1 (3.4)	25.5 (2.4)	26.5 (1.4)	<0.01	<0.01
PDQ total	31.1 (13.7)		25.5 (15.4)		0.05
FOG Questionnaire	9.2 (5.6)		6.6 (4.6)		0.01

Measures of gait, speech and quality of life are shown, as is total and sub-scores of the UPDRS parts 2 and 3 (off and on medication).

AIDS, Assessment of Intelligibility for Dysarthric Speech; A-LFS, acute low-frequency stimulation; HFS, high-frequency stimulation; LFS, low-frequency stimulation; FOG, freezing of gait; SA-LFS, sub-acute low-frequency stimulation; SWS, Stand–Walk–Sit; TMT, Tinetti Mobility Test; PDQ, Parkinson’s Disease Questionnaire 39; UPDRS, Unified Parkinson’s disease rating scale.

Case records of 168 patients fulfilling predetermined criteria were retrospectively reviewed. Of these, 96 patients tried LFS after the development of one (27 gait difficulties, 13 speech difficulties and 2 balance difficulties) or a combination (54 patients) of axial symptoms while 72 had remained on HFS throughout the defined study period. Patients’ pre-operative characteristics are summarized in Tables 2 and 3. Patients who were switched to LFS were (at the time of surgery), older (59.3 ± 8.4 versus 56.5 ± 9.4, *P* = 0.03), with less severe tremor (6.2 ± 5.7 versus 8.9 ± 6.7, *P* = 0.01) and a more severe axial burden (8.3 ± 3.6 versus 7.2 ± 3.6, *P* = 0.03), and had a shorter post-surgical latency to axial impairment (15.2 ± 13.7 months versus 22.4 ± 18.8 months, *P* ≤0.01) than those remaining on HFS.

**Table 2:** Demographic and clinical features prior to surgery of the patients who were switched to LFS or remained on HFS

	LFS patients (*n* = 96) Mean (SD)	HFS patients (*n* = 72) Mean (SD)	Univariate risk ratio (95% CI)	Multivariate risk ratio
Gender (M/F)	70/26	47/25		
Age at DBS, years	59.3 (8.4)*	56.5 (9.4)*	1.04 (1.01–1.06)*	1.01
Disease duration at DBS, years	12.8 (5.0)	12.4 (5.6)	0.51	0.51
Duration to first axial symptom, months	15.2 (13.7)*	22.4 (18.8)*	0.97 (0.96–0.98)*	0.97*
Baseline factors				
LEDD (mg/day)	1446.7 (633.9)	1293.9 (620.1)		
UPDRS-III Total (OFF)	44.4 (13.25)	46.3 (14.89)		
UPDRS-III Total (ON)	17.5 (8.9)	18.1 (8.9)		
UPDRS-III Tremor (OFF)	6.2 (5.7)*	8.9 (6.7)*	0.93 (0.90–0.97)*	0.94*
UPDRS-III Tremor (ON)	1.4 (2.5)*	2.7 (4.1)*	0.91 (0.84–0.98)*	1.00
UPDRS-III Axial (OFF)	8.3 (3.6)*	7.2 (3.6)*		
UPDRS-III Axial (ON)	3.1 (1.9)*	2.6 (1.8)*	1.12 (1.01–1.25)*	1.08
UPDRS-III Speech (OFF)	1.4 (0.9)	1.2 (0.8)		
UPDRS-III Speech (ON)	0.7 (0.8)	0.6 (0.7)		
UPDRS-III Gait (OFF)	2.2 (1.0)	1.9 (1.1)	1.22 (1.00–1.49)*	1.00
UPDRS-III Gait (ON)	0.7 (0.6)	0.6 (0.5)		
UPDRS-III Postural Stability (OFF)	1.5 (1.0)	1.3 (1.0)		
UPDRS-III Postural Stability (ON)	0.7 (0.6)	0.5 (0.6)		
UPDRS-II Speech (OFF)	1.6 (1.0)	1.6 (1.0)		
UPDRS-II Speech (ON)	0.7 (0.9)	0.6 (0.7)		
UPDRS-II Falls (OFF)	1.1 (1.1)	1.1 (1.1)		
UPDRS-II Falls (ON)	0.4 (0.7)	0.4 (0.7)		
UPDRS-II FOG (OFF)	1.9 (1.1)	1.4 (1.3)		
UPDRS-II FOG (ON)	0.6 (0.9)	0.4 (0.7)		
UPDRS-II Walking (OFF)	2.5 (1.0)	2.3 (1.0)		
UPDRS-II Walking (ON)	0.8 (0.9)	0.7 (0.8)		

Clinical characteristics and rating scales performed prior to surgery which predicted the need for low frequency stimulation on regression analysis are demarcated in columns 4 and 5. Variables with *P* < 0.05 were entered into the multivariate model.

FOG, freezing of gait; HFS, high-frequency stimulation; LFS, low-frequency stimulation; UPDRS, Unified Parkinson’s disease rating scale.

*P < 0.05.

**Table 3:** Clinical characteristics and rating scales which predicted a failure on low frequency stimulation with regression analysis

Variables	Mean scores of patients maintained on LFS (SD), *n* = 83 (% with available data)	Mean scores of patients reverted from LFS (SD), *n* = 13 (% with available data)	Univariate risk ratio (95% CI)	Multivariate risk ratio
Duration to first axial impairment onset (months)	13.5 (12.9)* (96%)	25.8 (14.2)* (100%)	1.06 (1.02–1.09)*	1.05*
Duration before switch to low frequency (months)	25.1 (21.4) (100%)	42.5 (26.1) (100%)	1.03 (1.01–1.04)*	1.00
Duration on low frequency (months)	33.0 (24.6) (100%)	6.0 (9.3) (100%)		
UPDRS 3 tremor OFF	5.7 (5.7)* (94%)	9.0 (5.5)* (100%)	1.09 (1.00–1.18)*	1.08
UPDRS 3 tremor ON	1.3 (2.4) (94%)	2.2 (3.3) (100%)	1.17 (0.08–1.41); *P* = 0.08	

Variables with *P* < 0.05 were entered into the multivariate model (age, gender, disease duration). *P < 0.05.

FOG, freezing of gait; HFS, high-frequency stimulation; LFS, low-frequency stimulation; UPDRS, unified Parkinson’s disease rating scale.

In multivariate analysis (adjusting for all variables reaching statistical significance as univariates): duration to first axial impairment occurring (beta = 0.97, *P* < 0.01) and the baseline UPDRS 3 tremor OFF score (beta = 0.94, *P* = 0.01), independently predicted the use of LFS (Table 2).

Of the 96 patients switched to LFS, 13 reverted back to HFS (7 worsening of appendicular symptoms, 3 loss of initial benefit and 3 no benefit). Patients had remained on LFS for a mean of 35.6 months when the study was conducted. Factors predicting failure of LFS included the duration to onset of axial impairments post DBS surgery, the duration prior to switching to LFS and the pre-surgical severity of tremor (UPDRS 3 tremor OFF). The duration to first axial impairments occurring (beta = 1.05, *P* = 0.03) remained an independent predictor of continuation of LFS on multivariate analysis (adjusting for all variables reaching statistical significance as univariates) (Table 3).

## Discussion

In this study, we were able to demonstrate objectively that in patients treated with high-frequency STN-DBS, switching to LFS can improve axial impairments, such as gait, postural stability and speech as well as UPDRS motor scores. While LFS has less beneficial effects than HFS for tremor, it can nevertheless lead to improvements in patient rated quality of life. We also confirm that not all patients switching to LFS have sustained improvements and we have identified a number of factors associated with the success and durability of LFS.

Our findings are in agreement with a number of other studies that report the benefits of LFS^7^^,^^8^ and may help explain why others have not always supported LFS use.^6^^,^^18^ In addition to the factors we identified, further possible explanations include differences in study design, size and duration, the LFS programming parameters (60 versus 80 Hz), and the degree of correction for TEED. Maintaining TEED is postulated to maintain beneficial motor effects alongside improving the specificity of stimulation of adjacent structures^5^^,^^8^ though this concept remains speculative.^19^ Our study included patients experiencing the whole range of axial impairments, while other studies have focussed purely on FOG^7^^,^^8^^,^^20–22^ or speech impairments.^5^^,^^7^ While we did not predetermine the presence of either FOG or speech impairment as inclusion criteria in our study, our objective assessments performed at the initial visit suggest that both of these phenomena can improve with LFS, in line with a number of previous studies suggesting these axial features may have shared adjacent anatomical pathways.^23^

Speech disturbances following STN DBS are heterogeneous and may relate to stage of Parkinson’s disease, medication, comorbidity as well as being induced by DBS itself.^23^ Our findings of improvements in intelligibility scores with LFS is reassuring and in agreement with some previous studies.^24^^,^^25^ HFS STN-DBS may result in a restricted articulatory range^26^ due to current spread to capsular fibres.^26^^,^^27^ While HFS attenuates STN neural synchrony broadly across the beta frequency band, LFS seems to be more selective, attenuating high beta power while amplifying alpha and low beta bands.^28^ From a practical perspective, these biological mechanisms^29^ possibly impact on the internal mapping of articulators and their afferent feedback, disrupting the co-ordination of articulatory, laryngeal and respiratory components. LFS possibly improves this co-ordination of phonation and respiration.^4^^,^^30^

Despite overall positive results, two patients were unable to tolerate LFS acutely. Although this occurred in a smaller proportion compared to other studies,^31^^,^^32^ this acute deterioration may reflect a subgroup where specific factors should be explored further. The most common reason for LFS intolerance reported include worsening of appendicular motor symptoms and more specifically tremor. While it is clear that this can in some instances limit the possible use of LFS, the majority of patients in our study were willing to continue on LFS and reported an overall improvement in quality of life despite the worsening of mean UPDRS 3 tremor sub-scores.

Although the majority of patients who were switched to LFS remained on this setting in the long term, 13.5% switched back to HFS within 6 months of trialling LFS. This observation is consistent with a previous report suggesting that patients who stay on LFS past the 1-year time point tend to remain on it for an extended period.^11^ Aside from tremor, failure of LFS is more likely to occur in those patients with later onset of axial features. This potentially indicates that disease progression rather than HFS-DBS is the predominant cause of axial features in these less responsive cases.

Our findings have a number of limitations. Firstly, the lack of a blinded comparator group limits our ability to exclude placebo effects which might have had an impact on the short-term outcomes, while the lack of systematic assessments beyond 15 days restricts the objectivity of the long-term effects of LFS. The retrospective nature of the larger cohort does provide real world data regarding the utility of LFS, however, and its larger size should be free from major systematic biases.

In conclusion, we found LFS to be of value in the management of axial symptoms which develop or worsen in patients treated with chronic HFS STN-DBS. Patients who develop these symptoms earlier after DBS and who suffer from less severe forms of tremor seem to respond best.

## Abbreviations

A-LFS =: acute low-frequency assessment
FOG =: freezing of gait
HFS =: high-frequency stimulation
LFS =: low-frequency stimulation
PDQ-39 =: Parkinson’s Disease Questionnaire 39
SA-LFS =: sub-acute low-frequency assessment
STN-DBS =: subthalamic nucleus deep brain stimulation
SWS =: Stand–Walk–Sit
TEED =: total electrical energy delivered
TMT =: Tinetti Mobility Test
UPDRS =: unified Parkinson’s disease rating scale
